# Ligand Size and Carbon-Chain Length Study of Silver Carboxylates in Focused Electron-Beam-Induced Deposition

**DOI:** 10.3390/nano13091516

**Published:** 2023-04-29

**Authors:** Jakub Jurczyk, Katja Höflich, Katarzyna Madajska, Luisa Berger, Leo Brockhuis, Thomas Edward James Edwards, Czesław Kapusta, Iwona B. Szymańska, Ivo Utke

**Affiliations:** 1 Laboratory for Mechanics of Materials and Nanostructures, Empa—Swiss Federal Laboratories for Materials Science and Technology, Feuerwerkerstrasse 39, 3602 Thun, Switzerlandthomas.edwards@empa.ch (T.E.J.E.); 2 Faculty of Physics and Applied Computer Science, AGH University of Krakow Al. Mickiewicza 30, 30-059 Kraków, Poland; 3 Helmholtz-Zentrum Berlin Für Materialien und Energie, Nanoscale Structures and Microscopic Analysis, Hahn-Meitner-Platz 1, 14109 Berlin, Germany; katja.hoeflich@helmholtz-berlin.de; 4 Ferdinand-Braun Institut, Leibniz-Institut für Höchstfrequenztechnik, Gustav-Kirchhoff-Str. 4, 12489 Berlin, Germany; 5 Faculty of Chemistry, Nicolaus Copernicus University in Toruń, Gagarina 7, 87-100 Toruń, Polandpola@umk.pl (I.B.S.)

**Keywords:** FEBID, nanoprinting, silver carboxylates

## Abstract

Gas-assisted focused electron-beam-induced deposition is a versatile tool for the direct writing of complex-shaped nanostructures with unprecedented shape fidelity and resolution. While the technique is well-established for various materials, the direct electron beam writing of silver is still in its infancy. Here, we examine and compare five different silver carboxylates, three perfluorinated: [Ag_2_(µ-O_2_CCF_3_)_2_], [Ag_2_(µ-O_2_CC_2_F_5_)_2_], and [Ag_2_(µ-O_2_CC_3_F_7_)_2_], and two containing branched substituents: [Ag_2_(µ-O_2_CCMe_2_Et)_2_] and [Ag_2_(µ-O_2_C^t^Bu)_2_], as potential precursors for focused electron-beam-induced deposition. All of the compounds show high sensitivity to electron dissociation and efficient dissociation of Ag-O bonds. The as-deposited materials have silver contents from 42 at.% to above 70 at.% and are composed of silver nano-crystals with impurities of carbon and fluorine between them. Precursors with the shortest carbon-fluorine chain ligands yield the highest silver contents. In addition, the deposited silver content depends on the balance of electron-induced ligand co-deposition and ligand desorption. For all of the tested compounds, low electron flux was related to high silver content. Our findings demonstrate that silver carboxylates constitute a promising group of precursors for gas-assisted focused electron beam writing of high silver content materials.

## 1. Introduction

Gas-assisted focused electron-beam-induced deposition (FEBID) is a direct method of nanoprinting nanostructures on any conductive substrate. It utilizes the electron beam of a scanning electron microscope (SEM) to directly deposit material in a desired shape. The gas injection system (GIS) delivers a gas of the precursor molecules from the reservoir, over the sample. The molecules adsorbed on the surface and are locally dissociated by the electron beam. The non-volatile parts chemisorb to the substrate, creating the deposit, and the volatile ones desorb and are pumped out of the chamber. In most of the cases, FEBID is used to deposit metals from organometallic precursors [1]. FEBID has been applied in many fields, including nano-magnetism [2,3,4], plasmonics [5,6,7,8] and photonics [9,10], the manufacturing of micro- and nano-sensors [11,12,13], single electron transistors [14] and memristors [15], and for atomic [16,17] and magnetic force microscopy tips [18], as well as for photolithography mask corrections [19]. 

The organometallic precursors for FEBID are, in most cases, volatile enough to be used with the GIS at room or moderately elevated temperatures (below 200 °C). The challenge remains to release the organic ligands from the metal atom centre and to nanoprint as pure metal structures as possible. Due to two unwanted mechanisms: the incomplete release of organic ligands (incomplete dissociation of all metal—ligand bonds) and the co-deposition of organic ligands (intra-ligand bond dissociation), the purity of typical as-deposited FEBID material from organometallic precursors is smaller than 40 at.% [20,21,22]. Residual hydrocarbons inside the vacuum chamber and on the substrate surface may also add carbon content. There are several methods of post-FEBID purification of the as-deposited material. Some of them involve e-beam irradiation in the presence of water vapour [23,24] or oxygen [17,25,26]. Others rely on heating either in a vacuum [27,28,29,30,31] or in a reactive atmosphere [32,33]. The challenge is to retain the shape of the deposited structure upon purification. Although there are some exceptions [5,34], in many cases, the shape of the deposited 3D structure was not preserved, mostly due to high volume reduction caused by the removal of the contaminant matrix from the as-deposited material. Only metal carbonyl precursors of cobalt and iron have been proven by several studies to reproducibly achieve high as-deposited metal content up to >90 at.%, while other high-metal content Au, Cu, and W FEBID materials did not find wide use due to precursors’ instability or handling issues of the (mostly inorganic) precursors [20,21,35]. Hence, it is of crucial importance to identify new FEBID compatible precursor molecules. Recently, the class of carboxylate compounds has been explored for the deposition of copper [33,36] and silver [37,38,39]. Carboxylates are known as chemical vapour deposition precursors, giving high purity thin layers of metal [40,41,42,43,44]. Even though oxygen atoms are directly bound to the metal atom, the direct electron beam writing with silver pentafluoropropionate ([Ag_2_(µ-O_2_CC_2_F_5_)_2_]) and silver 2,2-dimethylbutyrate ([Ag_2_(µ-O_2_CC(Me)_2_Et)_2_]) resulted in metal contents of about 75 at.% with almost no oxygen [37,39].

The mechanisms behind the large metal content obtained from [Ag_2_(µ-O_2_CC(Me)_2_Et_2_] and other carboxylates with aliphatic straight and branched carbon chains have been studied in condensed film electron-stimulated desorption experiments under ultra-high vacuum conditions. The ionization upon electron impact preferably removes charge from the carboxylate group leading with high efficiency to the formation of stable and volatile CO_2_ and an equally volatile alkene [45].

In view of these results, we present a comparative study of five silver carboxylates. Three perfluorinated compounds, which differ by the number of CF_2_ groups in their ligand carbon chains: [Ag_2_(µ-O_2_CCF_3_)_2_], [Ag_2_(µ-O_2_CC_2_F_5_)_2_], [Ag_2_(µ-O_2_CC_3_F_7_)_2_], and two non-fluorinated compounds differing by one CH_2_ group in their ligand: [Ag_2_(µ-O_2_C^t^Bu)_2_] and [Ag_2_(µ-O_2_CC(Me)_2_Et)_2_]. In our study we focus on the influence of the size and perfluorinated/non-fluorinated nature of the carboxylate ligand on the achieved metal content and structure of the FEB deposits.

## 2. Materials and Methods 

### 2.1. Chemical Formula and Properties of Silver Carboxylates

**Figure 1:**
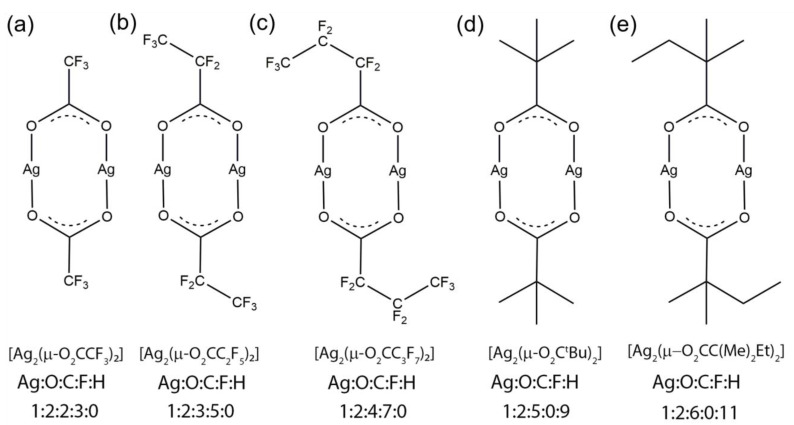
Structural formulae of studied silver precursors, sorted with increasing number of carbon atoms in ligands from left to right (**a**) silver trifluoroacetate, (**b**) silver pentafluoropropionate, (**c**) silver heptafluorobutyrate, (**d**) silver pivalate, (**e**) silver 2,2-dimethylbutyrate (or dimethylbutanoato-κO). The perfluorinated silver carboxylates form a series with carbon chain lengths of two to four carbon atoms and the non-fluorinated carboxylates continue with branched ligands containing five to six carbon atoms. The atomic ratios are noted for each precursor.

All of the investigated compounds have similar structural formulas with the silver atoms bonded to the oxygen atoms from the carboxylate ligand, see Figure 1. A dimeric structure containing bridges Ag-O-C(R)-O-Ag was observed in gas phase for all carboxylates [21,46,47,48]. 

All compounds are white crystalline powders that are stable in air and vacuum, which facilitates their use in any FEBID setup. The perfluorinated complexes were synthesised according to the process published by Szłyk et al. [49], non-fluorinated [Ag_2_(µ-O_2_C^t^Bu)_2_] according to Kuzmina et al. [47], and [Ag_2_(µ-O_2_CC(Me)_2_Et)_2_] according to Szymańska et al. [48]. The thermal properties of the silver carboxylates were previously investigated using vacuum thermal gravimetric analysis [50]. The main difference between fluorinated and non-fluorinated compounds is their thermal stability. Although the mass loss starts earlier for the latter (around 180 °C), they exhibit a significant amount of residues (even heated up in vacuum): around 45–50% of initial mass, suggesting simultaneous sublimation and thermal dissociation. This behaviour constrains their wide use in commercial GIS systems. In contrast, fluorinated precursors only showed <5 wt.% of mass residues in vacuum, indicating that sublimation dominates over thermal decomposition [50].

### 2.2. Precursor Delivery

All of the examined silver precursors have negligible room temperature volatility, in contrast to precursors typically used in FEBID for other metals. The carboxylate precursor molecules were delivered via a customized in-chamber stainless steel gas injection system (GIS) attached to a mobile three-axes holder. The GIS had a cylindrical nozzle of 3 mm diameter to provide enough precursor molecules over the sample substrate. The GIS temperatures for the silver carboxylate FEBID were around 150 to 190 °C, see Table 1, to establish a gas molecule impingement rate useful for FEBID. For comparison, typical precursors such as [Pt(η^5^-CpMe)Me_3_] (commonly referred to as Me_3_PtCpMe) for platinum require around 60 °C [22], while for cobalt FEBID with [Co_2_(CO)_8_], room temperature is already sufficient [27]. 

The molecule throughput *Q* through the GIS was determined by mass loss measurements for each precursor during the entire heating period using *Q* = (Δ*m*/Δ*t*)∙(*N_A_*/*M*), with measured mass loss Δ*m* during the time Δ*t* when the GIS was hot, *N_A_* the Avogadro number, and *M* the molecular mass of the silver carboxylate. As our GIS design did not include a valve, the supply of precursor was continuous throughout the entire heating period (usually a few hours per experiments). The molecule flux impinging at the substrate was simulated by our freeware GISsimulator [51,52]. It accounts for the nozzle geometry to map the impinging precursor distribution on the substrate, see Figure S1 in the supplementary information S1. The typical impinging molecule flux in the FEBID region was about 28% of the total flux exiting the nozzle outlet area. Table 1 gathers the GIS temperatures used and the related ranges of throughput, as well as the molecule flux of each silver carboxylate. However, no significant decrease in metal content for higher GIS temperature was noticed. 

### 2.3. Deposition Process

The FEBID experiments were performed using a Hitachi S3600 SEM equipped with a custom heating stage. The temperature of the stage was controlled via a thermocouple attached to a flat copper block clamped on a Boralectric resistance heater from Tectra (Frankfurt a M., Germany). The silicon substrates and silicon nitride membrane chips (for transmission electron microscopy) were clamped on top of the copper block. The heater was connected to a standard laboratory power supply. Pieces of Si<100> boron p-doped wafer with a native oxide layer were used as a substrate for deposit composition studies. Structures for transmission electron microscope (TEM) measurements were directly deposited on silicon TEM chips with open access windows to the 50-nm thick silicon nitride membrane coating (from TedPella, Redding, CA, USA). 

For each of the examined compounds, systematic optimization of thermal conditions of the deposition process was performed by increasing, in a stepwise manner, the stage and GIS temperatures. The maximum GIS temperature was then settled at the temperature where the flux of the molecules was sufficient for SEM visible deposition (5 min continuous spot exposures) while avoiding thermal decomposition of the precursor in the GIS reservoir. After the experiments, the leftover precursor from the GIS reservoir was weighed and visually examined. Any change in colour and structural appearance would indicate thermal decomposition of the compound. In case of visible changes, the GIS reservoir was completely emptied and cleaned before refilling. The measurement of the mass loss in such a case was discarded. Starting from the optimized GIS temperature, the lower limit of the stage (substrate) temperature was determined by gradually lowering it until condensation of the precursor on the substrate was observed by SEM (waiting time 30 to 60 min for 5 to 10 K steps of temperature difference between stage and GIS temperature). The upper temperature limit of the stage was determined as the temperature at which thermal decomposition of the precursor began to cover the entire sample surface. Finally, the substrate temperature was set in the upper half of this window to safely prevent condensation and CVD; i.e., each precursor requires its own optimized substrate temperature. Especially for the precursors [Ag_2_(µ-O_2_CC(Me)_2_Et)_2_], and [Ag_2_(µ-O_2_CC_2_F_5_)_2_], see Section 3.1 and Section 3.2 control of thermal decomposition was difficult during the FEBID experiment. 

All square deposits were carried out at an acceleration voltage of 20 kV (except for the one prepared with [Ag_2_(µ-O_2_CC_2_F_5_)_2_], which was made at 15 kV) and sample currents of 0.5–0.6 nA. Further deposition parameters of rectangular deposits were: 10 µm side length, 1 µs dwell time, 6 nm point pitch, and 400 passes using a spiral scan strategy from the outside to the centre. All spot deposits were made at the same acceleration voltage and the sample current using 5 min continuous exposure time. The beam diameter (full width at half maximum and full width containing 99.9% of electrons) was determined using the knife-edge method using the images of the lacy carbon films, as described in [37].

### 2.4. Characterization

The deposit morphologies were analysed using a Hitachi S4800 SEM. The compositions were measured with an energy dispersive X-ray spectroscopy (EDXS) system from EDAX. A voltage of 8 kV was chosen to provide enough intensity saturation for each measured characteristic X-ray line. The EDX signal was collected by scanning over square areas of around 1 × 1 µm^2^, which were positioned at least 1.5 µm away from the edge of the deposit, to avoid signal collection from Si not covered with the deposit. When the deposit was thin, the characteristic lines of the substrate atoms were visible. Quantification results for such spectra were corrected using the thin film SAMx StrataGEM software [53,54]. The EDX quantification was standardless. Residual hydrocarbons present in the vacuum chamber of the SEM impeded exact carbon quantification, meaning that all silver-to-carbon ratios and silver contents reported here can be taken as lower limit values. We measured carbon contamination build-up during EDX to be ≈15 at.% [38,39]. Room temperature condensate samples from silver precursors were also measured by EDX and confirmed silver content. Hence, we assumed that the GIS delivered intact gas phase molecules.

The thickness profiles and topography of all deposits were measured using an atomic force microscope (AFM) from NT-MDT in semi—contact (tapping) mode using standard Si Bruker tips RTESPA-300, see Figures S2.1–S2.10 in the Supplementary Information S2.

Transmission electron microscopy (TEM) structural characterization and diffraction measurements were performed in a JEOL JEM 2200 FS, operated at 200 kV in both high resolution (HR-TEM) and scanning mode (STEM). Electron transparent, 50-nm thick SiN_x_ membrane TEM grids were used. The data were analysed using CSpot software version 2.1.0.

Measurements of the resistance of FEBID line deposits by the four point probe method were made using a homemade setup described in [37,55].

## 3. Results and Discussion

### 3.1. Morphology and Composition of Square Deposits

**Figure 2:**
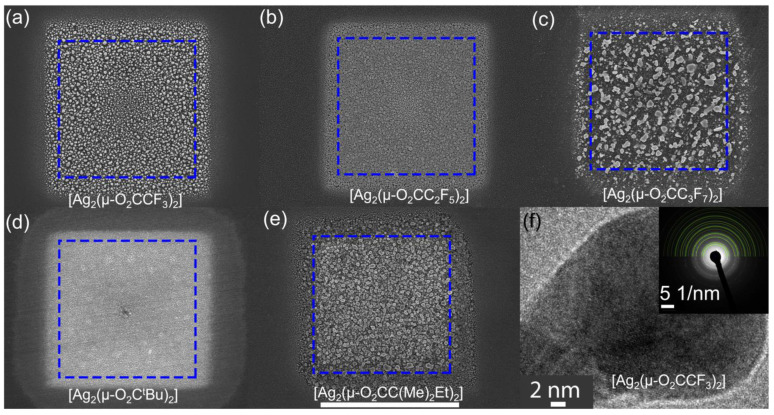
SEM top view images of FEBID square deposits of 10 × 10 µm^2^ from different silver carboxylates. The box (dashed blue line) represents the nominal size of the deposit. (**a**–**c**) show the fluorinated carboxylates, (**d**,**e**) show non-fluorinated carboxylates. The size of the scale bar is 10 µm. (**f**) HR-TEM image of an individual silver nanocrystal obtained by FEBID with [Ag_2_(µ-O_2_CCF_3_)_2_]. Comparable nanocrystals were also observed for [Ag_2_(O_2_CC_3_F_7_)_2_] and [Ag_2_(µ-O_2_CC(Me)_2_Et)_2_]. The diffraction inset indicates pure silver by indexing.

Figure 2a–e shows SEM images of squares deposited with the different precursors: (a) [Ag_2_(µ-O_2_CCF_3_)_2_], (b) [Ag_2_(µ-O_2_CC_2_F_5_)_2_], (c) [Ag_2_(µ-O_2_CC_3_F_7_)_2_], (d) [Ag_2_(µ-O_2_C^t^Bu)_2_], (e) [Ag_2_(µ-O_2_CC(Me)_2_Et)_2_]. The morphology of the square deposits is granular with a rough surface. The growth rates were estimated based on the average thickness of the deposits determined by AFM (see Supplementary Information S2.2, Figures S2.6–S2.10) and on the deposition time. The values span from 0.9 Å/s ($0.9\times 10^{-2}\;{\mu m}^{3}/s)$ for both [Ag_2_(O_2_CC_3_F_7_)_2_] and [Ag_2_(µ-O_2_CC(Me)_2_Et)_2_], through 1.4 Å/s ($2.3\times 10^{-2}\;{\mu m}^{3}/s)$ for [Ag_2_(µ-O_2_CCF_3_)_2_], 2.1 Å/s for [Ag_2_(µ-O_2_C^t^Bu)_2_] ($2.4\times 10^{-2}\;{\mu m}^{3}/s)$, and up to 2.8 Å/s for [Ag_2_(µ-O_2_CC_2_F_5_)_2_] ($4.7\times 10^{-2}\;{\mu m}^{3}/s)$. There was no correlation of the growth rate with the number of carbon atoms and the chain or branch type of the ligands in our experimental conditions. The highest growth rate was obtained for [Ag_2_(µ-O_2_CC_2_F_5_)_2_], the only one for which three-dimensional growth had previously been reported for [38]. We also obtained a vertical FEBID pillar with [Ag_2_(µ-O_2_CCF_3_)_2_], see Supplementary Information S3. However, we had difficulties in reproducing it probably due to a very narrow temperature window for this precursor [55]. Blue dashed lines show the nominal 10 × 10 µm^2^ deposit area, programmed with the patterning software. The actual obtained side length of each deposited square was visibly larger than 10 µm. This relates to the size of the electron beam, which was determined to be around 450 nm at full beam width containing 99% of electrons. Moreover, in all previously reported cases, deposits made with silver carboxylates had significant halos—material deposited around the actual irradiation spot, due to interactions of secondary electrons of second type (SE II) with adsorbed precursor molecules [30,31,40]. A more detailed discussion regarding halos can be found in the next section of this study. 

**Figure 3:**
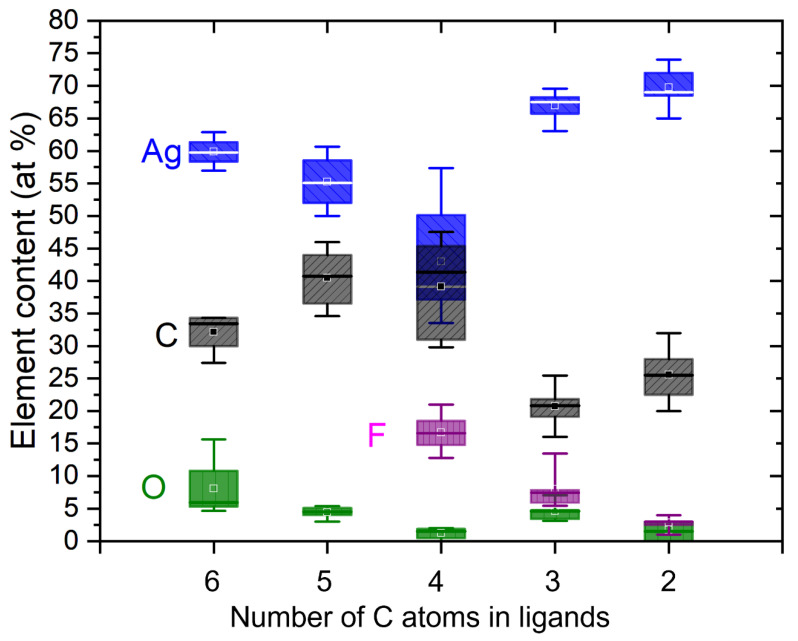
Box plot showing the median value (solid horizontal line), mean value (square), the quartile box, and the minimum and maximum values of compositions measured on at least four FEBID square deposits from each precursor indicated by the number of carbon atoms contained in the ligand of the pristine precursor molecule, see Figure 1. Values are not corrected for carbon contamination build-up during EDX, see Section 2.4.

EDX measurements show that all carboxylate-derived deposits can provide a maximum silver content above 50 at.%. The highest value of 74–76 at.% Ag was achieved using [Ag_2_(µ-O_2_CCF_3_)_2_]. For each silver carboxylate precursor, at least four FEBID squares with identical patterning parameters and electron beam conditions, their average and best-of values are listed in Table 2 and their statistics are illustrated in Figure 3. Notably, we did not subtract the carbon content related to contamination writing during EDX data acquisition, which was determined to be 15 at.% in earlier studies [39].

The most interesting observation from Table 2 is the low oxygen content in all deposits. The Ag:O ratio changes from 1:2 present in the precursor molecule to values between 1:0.02 and 1:0.1 in the deposit. This indicates the dominant dissociation of silver-oxygen bonds in all silver carboxylates, most probably due to the formation of stable and volatile CO_2_ [45], which efficiently removes oxygen from the pristine adsorbate. After dissociation, the reaction products reside on the surface until they desorb or until they further dissociate by successive electrons into non-volatile carbon and mostly volatile C_y_H_x_ or C_y_F_x_ fragments. In this respect, the Ag:C ratio is informative. According to Table 2, for example, the carbon content in the measured best-of purity deposits drops to about 9–14% of its initial stoichiometric value in the corresponding precursor molecule, indicating an efficient ligand release and desorption. This is in line with the decrease in the measured fluorine content to roughly 1% to 3% of the initial fluorine content in the precursor. As shown in Table 2, the precursor with the longest fluorinated carbon chain [Ag_2_(µ-O_2_CC_3_F_7_)_2_] has a deposit composition of CF_0.5_ in the remaining organic residue, while for the silver carboxylates with a shorter fluorinated carbon chain, the organic residue is mainly carbon, doped with fluorine at a much lower concentration. Figure 3 shows the boxplot statistics of the absolute atomic percentages of all the FEB deposits investigated. The highest metal content for the perfluorinated silver carboxylates (both average and best recorded) was observed for the shortest chain ligand O_2_CCF_3_ (precursor of Figure 1a) and decreased when CF_2_ groups were added to the ligand (precursors of Figure 1b,c). The smallest silver content and the largest scatter in the silver and carbon content was observed for the fluorinated [Ag_2_(µ-O_2_CC_3_F_7_)_2_] precursor with four carbons per silver in the compound. However, these trends reverse for non-fluorinated compounds containing five and six carbon atoms. 

To confirm the presence of elemental silver, transmission electron microscope measurements were performed on a series of spot and line deposits on SiN_x_ membranes. Figure 2f depicts a representative HRTEM image of grains from a spot deposit made with [Ag_2_(µ-O_2_CCF_3_)_2_], showing parallel atomic planes indicating crystallinity. Diffraction patterns, on both line and spot deposits from all precursors, fit the pattern of pure silver, proving that no silver oxide was formed during FEBID for all of the precursors studied. However, in the case of [Ag_2_(µ-O_2_C^t^Bu)_2_] and [A_g2_(µ-O_2_C_2_F_5_)_2_], there was a peak, which could be associated with Ag_2_O (220). It is then possible, that for these precursors, there can be a mixture of silver and silver oxide in the deposit. Further diffraction patterns for deposits made with all silver carboxylates are available in the Supplementary Information S4. These TEM data demonstrate that deposition using the silver carboxylates with the electron beam mostly yields pure silver crystals in the deposit.

Our characterization results confirm that silver carboxylates provide the exciting prospect of realizing high-metal-content deposits of silver in gas-assisted FEBID that were otherwise only achievable using liquid phase silver FEBID [56]. There are several reasons for the high silver metal content deposition. First, all silver carboxylates seem to have silver-oxygen bonds, being very sensitive to dissociation by electron irradiation [38]. Second, the electron-induced fragmentation can occur under loss of CO_2_, and an alkene providing stable and volatile reaction products for efficient desorption as was shown in [45]. Third, the heated substrate drives surface diffusion and enhances desorption of adsorbed volatile molecules and fragments. This can reduce the overall efficiency of deposition by causing small residence times of precursor molecules [57], but it also increases the desorption rate of released ligands and thus avoids the co-deposition of carbon by further electron-induced ligand dissociation. Finally, the surface diffusion of metal atoms and small clusters results in the growth of large silver metal grains due to Ostwald ripening [58].

### 3.2. Morphology and Composition of Spot Deposits

**Figure 4:**
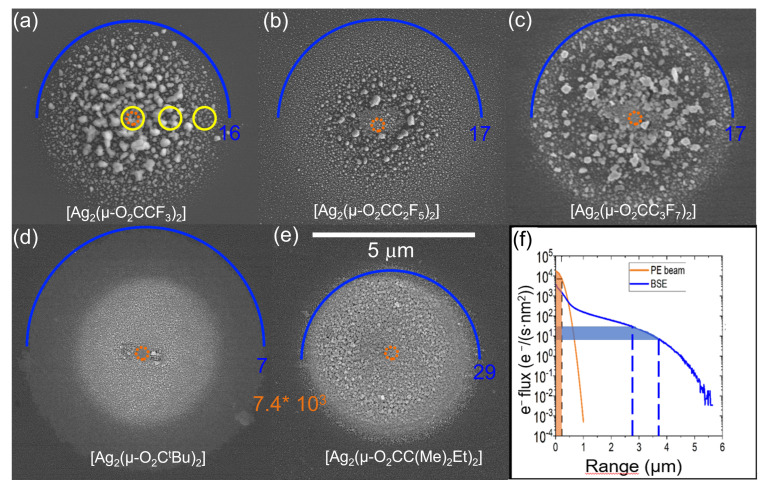
(**a**–**e**) SEM top view images of FEBID spots. Irradiation was with 20 kV for 5 min using different silver carboxylates, top: fluorinated silver carboxylates and bottom non-fluorinated silver carboxylates. Precursors are indicated. The primary electron beam (FW99) and the visible halo periphery are indicated by orange circles and blue half circles, respectively. Correspondingly coloured electron flux values in e^−^/(nm^2^s) were calculated from the Monte Carlo simulated BSE profile in (**f**). The blue band in (**f**) comprises the maximum extensions of the halo regions and its corresponding flux ranges for deposits made with all of the compounds. The dashed line corresponds to FW(99.9%)/2 of electrons. Yellow circles indicate the spots were EDX measurements were taken. The size of the circles corresponds to the maximum EDX excitation range. Note that there was a slight drift in (**d**) during FEBID.

To further study the deposition process with respect to the balance of arriving molecules and electrons, spot depositions were performed with all silver carboxylates. The common feature for all carboxylate deposits in Figure 4 is a visible amount of material deposited in the halo region. The spot deposits enable a combinatorial investigation of deposit purities as functions of the electron flux, as it decreases orders of magnitude from the centre to the halo periphery. For [Ag_2_(O_2_CC_3_F_7_)_2_] and [Ag_2_(µ-O_2_CC(Me)_2_Et)_2_], it was hard to distinguish the halo region from the central part of the deposit in the deposit thickness profiles (cf. Supplementary Information, Figures S2.1–S2.5) indicating a pronounced precursor-limited FEBID regime. For only three precursors, [Ag_2_(µ-O_2_C^t^Bu)_2_], [Ag_2_(µ-O_2_CCF_3_)_2_], and [Ag_2_(µ-O_2_CC_2_F_5_)_2_], the centre was visibly thicker than the halo. 

The halo is created due to the dissociation of adsorbed precursor molecules by secondary electrons generated by backscattered electrons (BSE) and the BSE electrons themselves. The orange, dotted ring in the middle of each deposit represents the calculated beam size at full width containing 99% of primary electrons, which was around 450 nm. Monte Carlo simulations [59] of 20 keV electrons vertically impinging on silicon, taking into account the primary electron beam size, resulted in the radial BSE flux profile shown in Figure 4f, which allowed the assigning of electron flux values to the outermost SEM-visible halo circumferences (marked for each deposit in Figure 4a–e). The order of magnitude of the electron flux at the visible halo circumference varied from 7 to 29 e/(nm^2^s), see Figure 4, and is a measure of the electron sensitivity towards the dissociation of the silver precursors. The existence of distinctly visible halos points to a high electron sensitivity of silver carboxylates in comparison to other organometallic precursors, for which such halos are not seen.

The morphology of the halo significantly differs between different carboxylates. The deposits made using non-fluorinated carboxylates results in more homogeneous halo regions, in which the grain sizes do not differ much along the halo radii. On the other hand, for perfluorinated species, the particle sizes within the halo region decrease with increasing distance from the centre. All deposits have in common that grains of deposited material are small in the primary beam region of highest lateral electron density, and that the silver content is higher in the halo region. This is in line with our previous studies on [Ag_2_(µ-O_2_CC_2_F_5_)_2_] [37] and [Ag_2_(µ-O_2_CC(Me)_2_Et)_2_] [39]. 

As the electron flux exponentially decreases from the centre to the outside, the maximum possible dissociation rate, being the product of the dissociation cross section and the radial electron flux, does so too. In other words, the FEBID regimes radially change from the centre to the outside from mass-transport (adsorbate) limited to desorption dominated or reaction rate (electron) limited. The corresponding implications on the FEB deposition rate were studied by Sanz-Hernandez et al. [60]. The adsorbate limited regime in the central region is responsible for the low growth rate despite the intense electron flux, especially due to the large adsorbate desorption rate caused by the elevated stage temperatures, which entails low adsorbate coverage. Furthermore, the strong depletion of intact precursor adsorbates by dissociation within the halo region reduces their supply by surface diffusion into the central region of the deposit. As a result, adsorbate replenishment is limited to impingement from the gas phase (from the GIS) in the central region. Another consequence of the changing FEBID regime is the possible change in composition. Based on FEBID experiments with [Ag_2_(µ-O_2_CC(Me)_2_Et)_2_], it has been proposed that, in the centre of the beam, the released carboxylate ligands are co-dissociated into non-volatile carbon fragments as there their desorption rate is low compared to their dissociation rate [30]. At larger radial distances—in the halo region—the relation of these rates inverses and the ligands have enough time to desorb before they are co-deposited [30,31]. This explains the lower silver content in the centre with respect to the halo; see Table 3. The silver-rich halo deposition can also explain why, for [Ag_2_(µ-O_2_CC_2_F_5_)_2_], [Ag_2_(µ-O_2_CC(Me)_2_Et)_2_], and [Ag_2_(µ-O_2_C^t^Bu)_2_], the silver content in the centre of spot deposits is lower than the average silver content measured in the square deposits (compare Figure 3 and Table 2). For the square deposits, all of the edge pixel exposures contribute with silver-rich halos towards the square centre. Furthermore, the silver-rich halo conditions suggest that low pixel exposure times in combination with sufficient non-irradiation time between the pixel exposures would also allow for the efficient desorption of volatile fragments and lead to silver-rich material inside the beam centre [39]. Approaching the periphery regions, the silver content in the spot deposit halos decreases again, probably because the electron flux is too low to fully dissociate the precursor adsorbates. It is worth noting that we could reproduce the Ag content in the centre of spot deposits for the [Ag_2_(µ-O_2_CC_2_F_5_)_2_] achieved by Berger et al. [37]. Finally, the oxygen content in the spot deposits is low (<7 at.%) and insignificantly varies with the electron flux (except for the case of [Ag_2_(µ-O_2_C^t^Bu)_2_]), so that we can exclude the oxidation of silver grains in the presence of residual oxygen or reduction of (intermediate) silver oxides as observed in a transmission electron microscope with silver foil and residual gas [61].

Finally, we observed that the absolute silver grain sizes vary between the different precursors. In the case of silver carboxylate FEBID, the substrate temperatures are in a range where the metal clusters become mobile, can coalesce, and increase in size due to Ostwald ripening [58]. We hypothesize that the individual carbon/fluorine/oxygen residues of each precursor influence these mechanisms. These mechanisms do not contribute to the metal content of the deposited material, but may significantly reduce the achievable shape fidelities and influence the percolation of deposited material. The percolation of metal grains in FEBID-grown gold line deposits is known to highly influence the electrical resistivity of the deposited material [62]. The best room temperature resistivity benchmark value we obtained so far was 21 μΩcm for [Ag_2_(µ-O_2_CCF_3_)_2_] [55]. This value can be improved in future by achieving better percolation of the silver grains, but was not within the scope of this study.

## 4. Conclusions

A comparative study of silver deposition with a focused electron beam was carried out using five different carboxylates, of which three were perfluorinated: [Ag_2_(µ-O_2_CC_3_F_7_)_2_], [Ag_2_(µ-O_2_CC_2_F_5_)_2_], and [Ag_2_(µ-O_2_CCF_3_)_2_] with straight-chain ligands, and two were non-fluorinated: [Ag_2_(µ-O_2_CC(Me)_2_Et)_2_] and [Ag_2_(µ-O_2_C^t^Bu)_2_] with branched ligands. All of the compounds were very sensitive to electron impact, as witnessed by visible halo deposition. The deposits consist of elemental silver grains with carbonaceous residues with low amounts of fluorine and oxygen distributed between these metal grains. The precursors have variations in ligand chains and branching, yet still the removal of non-volatile carbon atoms was very efficient, as measured from the composition of the as-deposited materials. Particularly, the removal of fluorine was close to 100%, reaching this value for the spot deposit with [Ag_2_(µ-O_2_CCF_3_)_2_]. Only the longest perfluorinated carbon chain precursor, [Ag_2_(µ-O_2_CC_3_F_7_)_2_], showed relatively low 43 at.% Ag content on average. The other silver carboxylates resulted in higher average silver contents, from >55 at.% for [Ag_2_(µ-O_2_C^t^Bu)_2_] to >70 at.% for [Ag_2_(µ-O_2_CCF_3_)_2_]. The highest silver contents were obtained for the compounds with the shortest perfluorinated chains. However, the high evaporation temperatures of perfluorinated carboxylate precursors require high substrate temperatures to avoid condensation. Consequently, the FEBID process could be considered as thermally assisted. While this helps to achieve high-metal-content deposits through the enhanced desorption of released ligands, it also reduces the residence time of the pristine adsorbates, leading to pronounced halo deposition and a low vertical growth rate in the beam centre. In addition, the structural reorganization due to the high surface mobility of silver atoms complicates the structural fidelity that can be achieved. Short pixel exposure times in combination with sufficient non-irradiation time have been suggested to obtain silver-rich deposit material inside the focused primary beam. Then, halo deposition could be reduced in future using low primary electron energy to achieve well-defined silver nanostructures. However, high substrate temperatures can increase the risk of non-selective silver deposition by thermal decomposition. Therefore, silver precursors that combine efficient decomposition channels that transform the ligands into stable, quickly desorbing, volatile products with lowest possible processing temperatures to minimize surface diffusion of the deposited silver atoms are still highly desirable. In summary, this study demonstrates the potential and challenges of carboxylates for the generation of high purity FEBID silver deposits and points out ways forward for the development of further precursors.

## Figures and Tables

**Table 1:** Summary of ranges of gas injection system (GIS) temperatures, molecule throughput *Q* through the heated GIS and impinging molecule flux *J* on the substrate at FEBID position for the silver carboxylates with the molar mass *M*.

Precursor	GIS (°C)	*Q* (10^14^ Molecules/s)	*J* (10^15^ Molecules/(s·cm^2^))	*M* (g/mol)
[Ag_2_(µ-O_2_CCF_3_)_2_]	170–185 °C	2.8–2.9	1.1–1.2	221
[Ag_2_(µ-O_2_CC_2_F_5_)_2_]	150–180 °C	3.8–5.2	1.4–2.0	271
[Ag_2_(µ-O_2_CC_3_F_7_)_2_]	175–190 °C	4.4–6.0	1.7–2.4	321
[Ag_2_(µ-O_2_C^t^Bu)_2_]	170–190 °C	2.2–4.9	0.9–2.0	209
[Ag_2_(µ-O_2_CC(Me)_2_Et)_2_]	145–175 °C	0.2–11.0	0.1–4.5	222

**Table 2:** Summary of stoichiometry of examined silver carboxylates in comparison to average and best-of atomic ratio and the best and average achieved metal content in deposited square structures. As no correction was performed for carbon contamination build-up during EDX, see Section 2.4, we noted Ag contents with the >sign.

Precursor	Precursor Stoichiometry Ag:O:C:F:H	Atomic Ratio of Best Purity Ag:O:C:F	Best Ag (at.%)	Atomic Ratio Average Ag:O:C:F	Average Ag (at.%)
[Ag_2_(µ-O_2_CCF_3_)_2_]	1:2:2:3:0	1:0.02:0.29:0.03	>74	1:0.03:0.36:0.04	>70
[Ag_2_(µ-O_2_CC_2_F_5_)_2_]	1:2:3:5:0	1:0.04:0.27:0.11	>70	1:0.08:0.41:0.10	>66
[Ag_2_(µ-O_2_CC_3_F_7_)_2_]	1:2:4:7:0	1:0:0.52:0.22	>57	1:0.02:0.91:0.40	>43
[Ag_2_(µ-O_2_C^t^Bu)_2_]	1:2:5:0:9	1:0.08:0.56:0	>61	1:0.07:0.75:0	>55
[Ag_2_(µ-O_2_CC(Me)_2_Et)_2_]	1:2:6:0:11	1:0.08:0.51:0	>63	1:0.12:0.60:0	>58

**Table 3:** Summary of atomic ratios of spot deposits measured by EDX in three places, see Figure 4. Values are not corrected for carbon contamination build-up during EDX, see Section 2.4.

Ag:O:C:F	Centre	Halo	Periphery
[Ag_2_(µ-O_2_CCF_3_)_2_]	73:0:27:0	76:0:24:0	56:0:43:1
[Ag_2_(µ-O_2_CC_2_F_5_)_2_]	45:1:51:3	46:0:51:3	35:1:58:6
[Ag_2_(µ-O_2_CC_3_F_7_)_2_]	50:0:49:1	60:0:39:1	58:0:41:1
[Ag_2_(µ-O_2_C^t^Bu)_2_]	38:7:55:0	65:2:33:0	43:12:45:0
[Ag_2_(µ-O_2_CC(Me)_2_Et)_2_]	42:7:51:0 *	70:7:23:0 *	---

* Values taken from [39].

## Data Availability

’The data is available on reasonable request from the first author.

## Supplementary Materials

The following are available online at https://www.mdpi.com/article/10.3390/nano13091516/s1, S1. Distribution of the impinging flux of precursor molecules as a fraction of the flux leaving nozzle exit; S2. AFM of spot and square deposits, S3. 3D structure made with [Ag_2_(µ-O_2_CCF_3_)_2_], S4. Integrated SAED patterns, marked and fitted to pure Ag pattern. Reference [63] is cited in the Supplementary Materials.
