# Supplementary material for: Ligand Size and Carbon-Chain Length Study of Silver Carboxylates in Focused Electron-Beam-Induced Deposition
Source: Nanomaterials (Basel). 2023 Apr 29;13(9):1516. doi: 10.3390/nano13091516 (PMC10180361; doi:10.3390/nano13091516)
Supplement: Supplementary file 1 [file nanomaterials-13-01516-s001.zip › nanomaterials-2322700-supplementary.pdf]

Supporting information to:

Chain length study of silver carboxylates in focused electron beam induced deposition

Jakub Jurczyk[1,2†*], Katja Höflich[3,4], Katarzyna Madajska[5], Luisa Berger[1], Leo Brockhuis[1,2], Thomas Edward James. Edwards[1], Czesław Kapusta[2], Iwona B. Szymanska[5], Ivo Utke[1*]

S1. Distribution of the impinging flux of precursor molecules as a fraction of the flux leaving nozzle exit

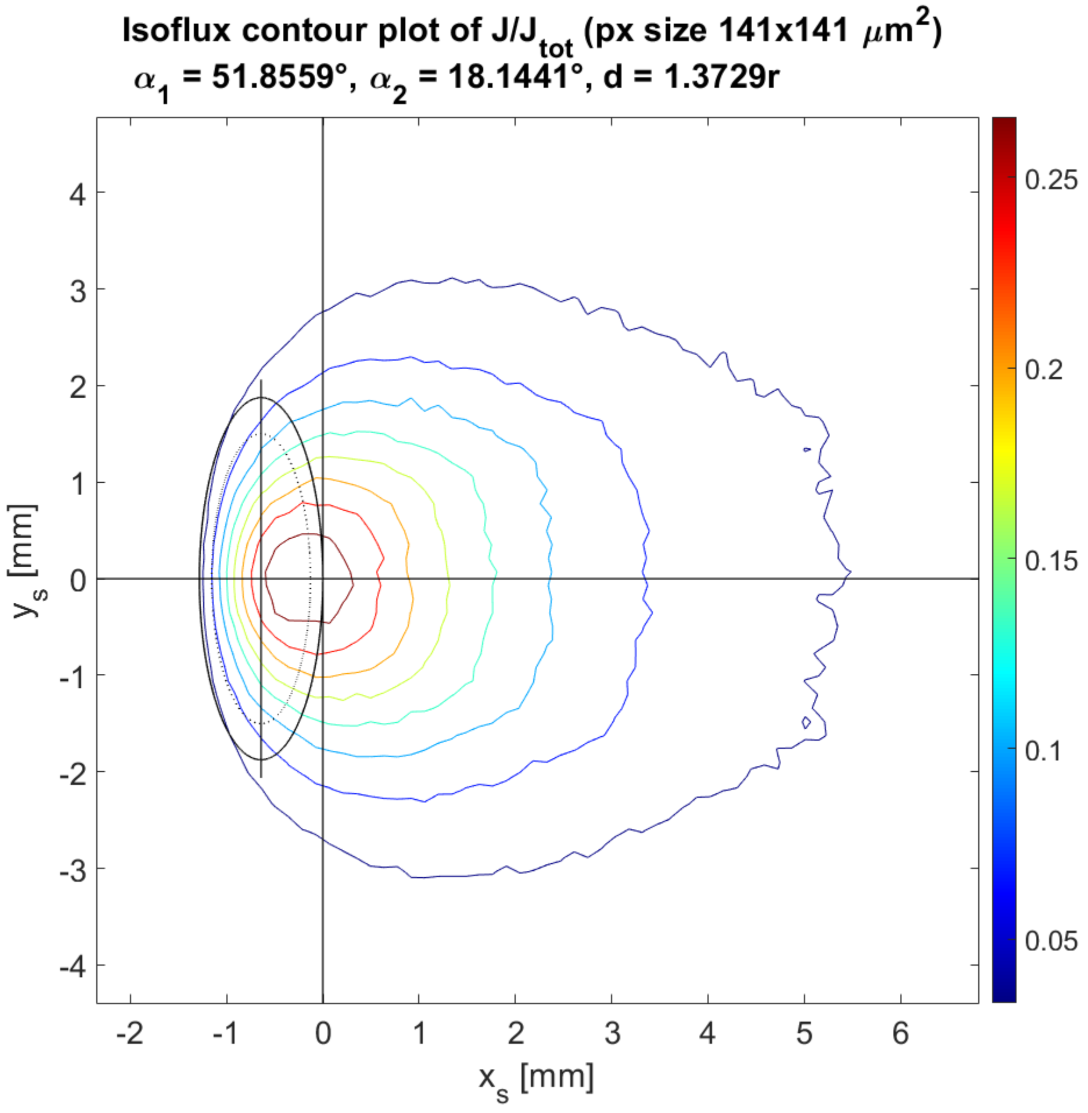

Figure S1. Distribution of the impinging flux of molecules as a fraction of the flux leaving nozzle exit.

All presented FEBID experiments were performed at around 0.2 mm of lateral distance from the upper edge of the nozzle exit, at 0 position on the x-axis.

S2. AFM of the spot and square deposits

The AFM profiles were measured using NT-MDT atomic force microscope in tapping mode with RTESPA 300 tips. The data was analysed using Gwyddion software and plotted using Origin 2022. All AFM profiles cross the centers of the spot and square deposits.

S2.1. AFM of spot deposits

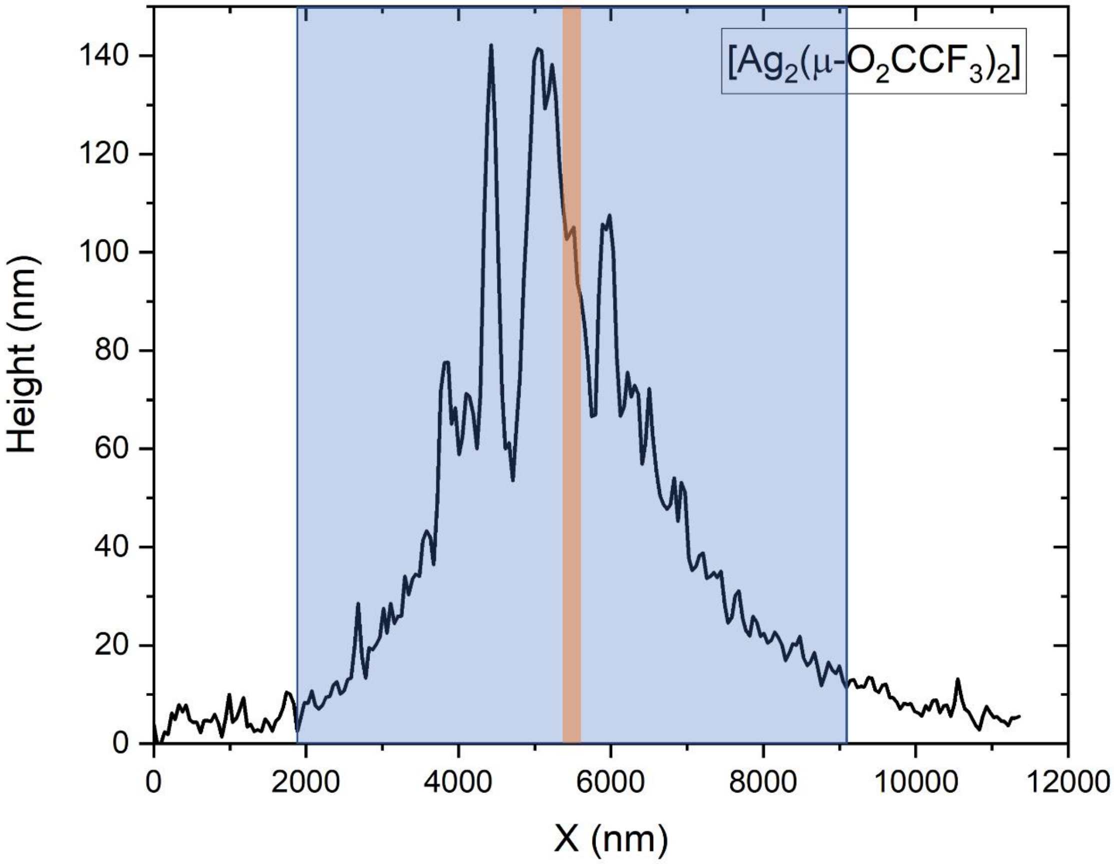

Figure S2.1 AFM profile of the spot deposit made with $[Ag_2(\mu\text{-}O_2CCF_3)_2]$, see fig. 4a of the manuscript. Orange rectangle indicates the FW99.9% range of the primary electron beam. The blue rectangle indicates the range of halo.

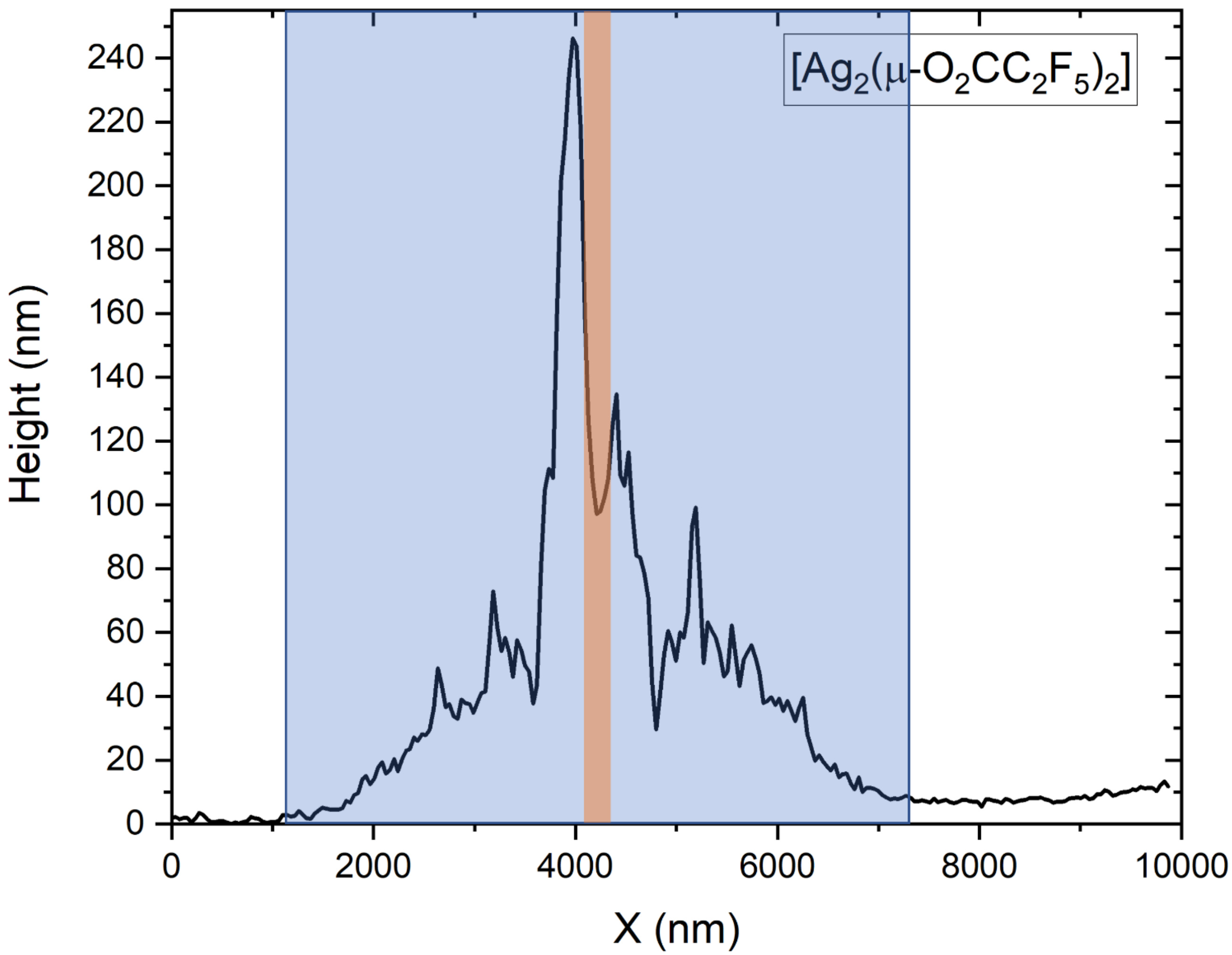

Figure S2.2 AFM profile of the spot deposit made with $[Ag_2(\mu\text{-}O_2CC_2F_5)_2]$, see fig. 4b of the manuscript. Orange rectangle indicates the FW99.9% range of the primary electron beam. The blue rectangle indicates the range of halo.

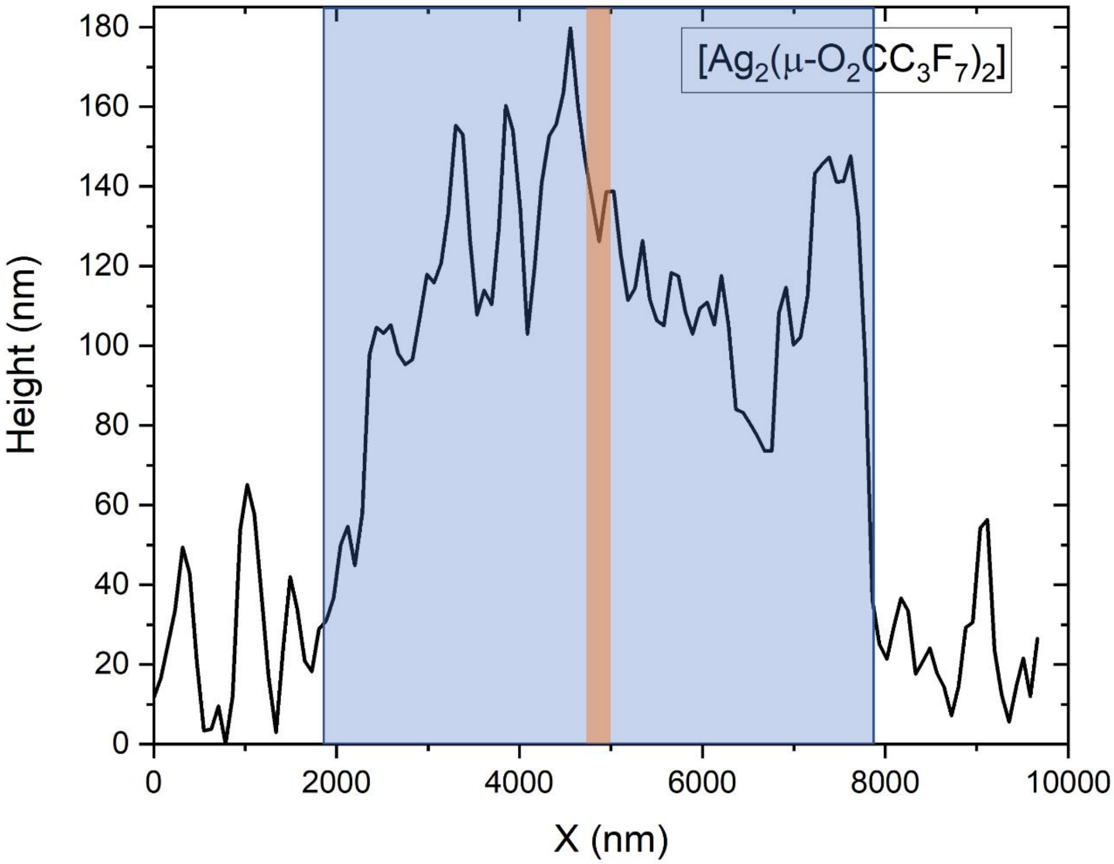

Figure S2.3 AFM profile of the spot deposit made with [$Ag_2(\mu\text{-}O_2CC_3F_7)_2$], see fig. 4c of the manuscript. Orange rectangle indicates the FW99.9% range of the primary electron beam. The blue rectangle indicates the range of halo.

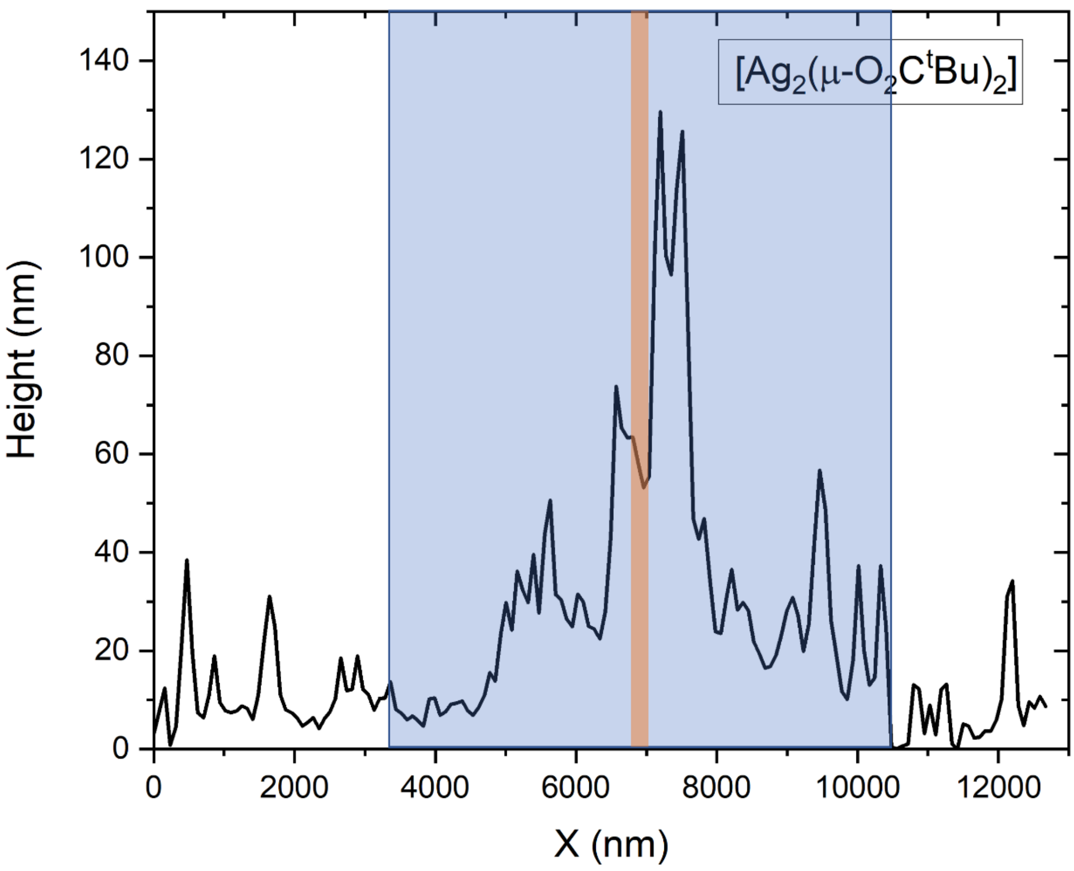

Figure S2.4 AFM profile of the spot deposit made with [Ag$_2$(μ-O$_2$C^{t}Bu)$_2$], see fig. 4d of the manuscript. Orange rectangle indicates the FW99.9% range of the primary electron beam. The blue rectangle indicates the range of halo.

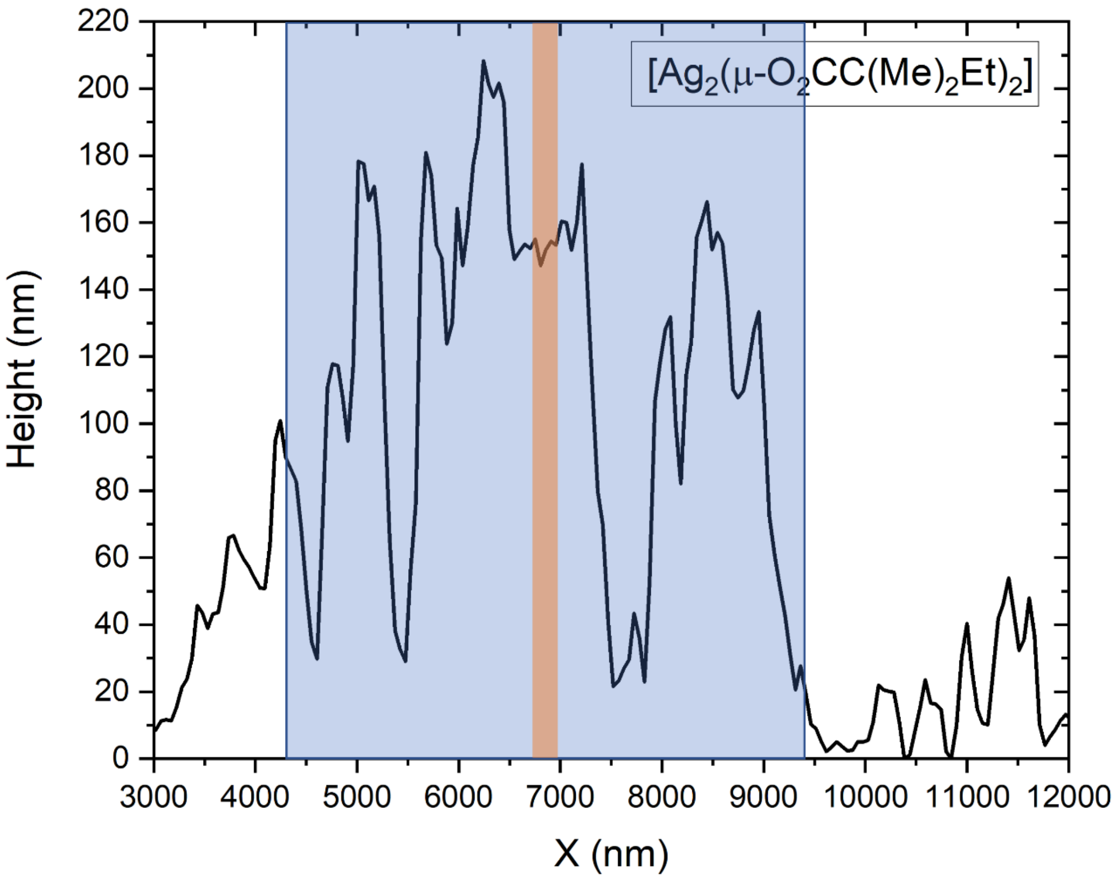

Figure S2.5 AFM profile of the spot deposit made with [$Ag_2(\mu\text{-}O_2CC(Me)_2Et)_2$], see fig. 4e in the manuscript. Orange rectangle indicates the FW99.9% range of the primary electron beam. The blue rectangle indicates the range of halo.

S2.2. AFM of square deposits

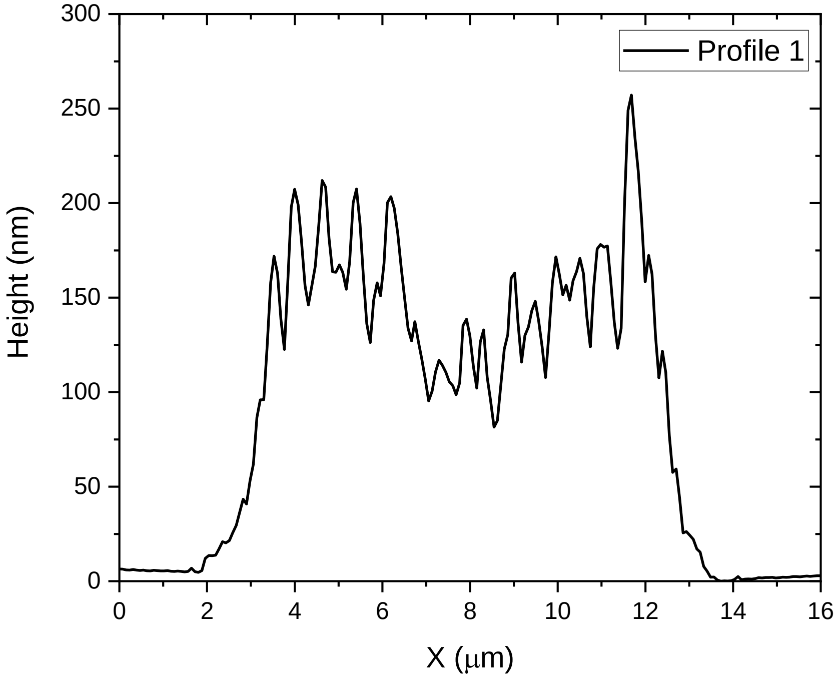

Figure S2.6 AFM profile of the 10×10 μm² (nominal size) square FEB induced deposit with $[Ag_2(\mu\text{-}O_2CCF_3)_2]$.

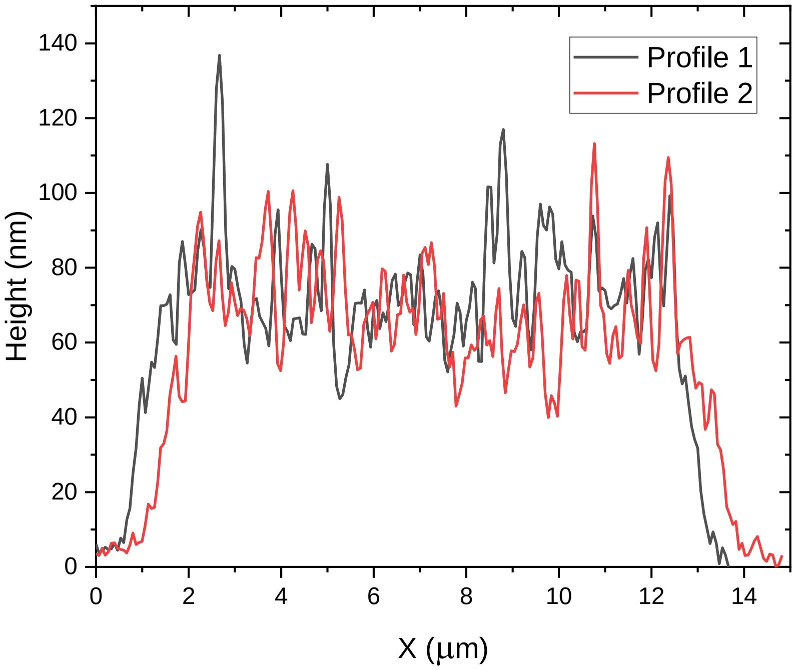

Figure S2.7 AFM profile of the 10×10 μm² (nominal size) square FEB induced deposit with $[Ag_2(\mu\text{-}O_2CC_2F_5)_2]$.

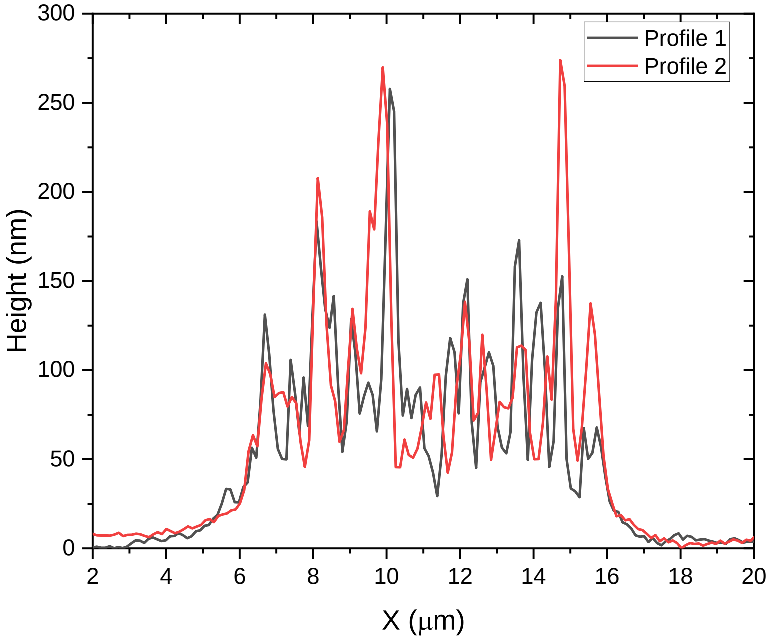

Figure S2.8 AFM profile of the 10×10 μm² (nominal size) square FEB induced deposit with [Ag_2(μ-$O_2CC_3F_7$)$_2$].

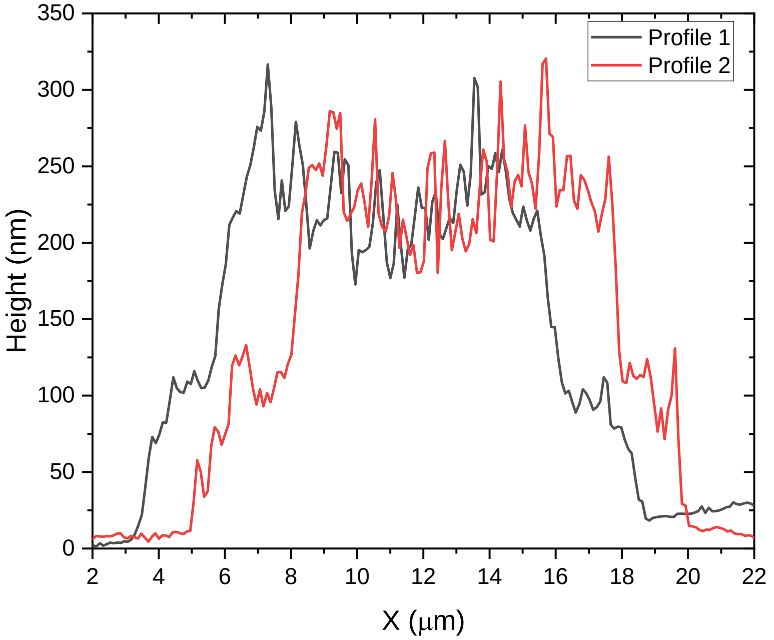

Figure S2.9 AFM profile of the 10×10 μm² (nominal size) square FEB induced deposit with [Ag_2(μ-O_2CC^tBu)$_2$].

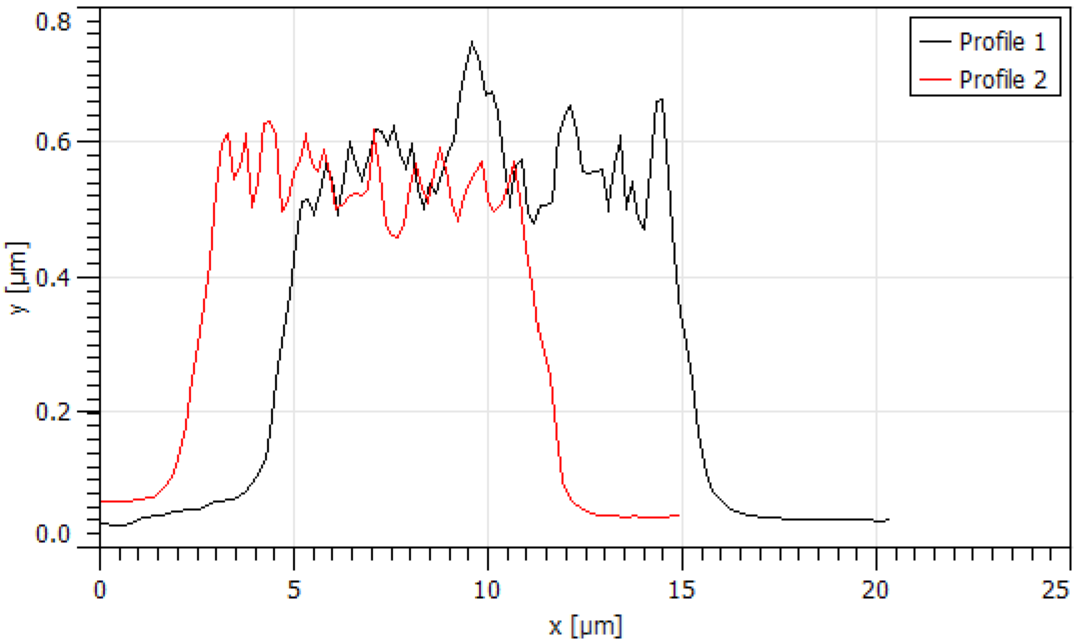

Figure S2.10. AFM profile of the 10×10 µm² (nominal size) square FEB induced deposit with [Ag_2(µ-$O_2CC(Me)_2Et)_2$].

S3. 3D structure made with [$Ag_2(\mu\text{-}O_2CCF_3)_2$].

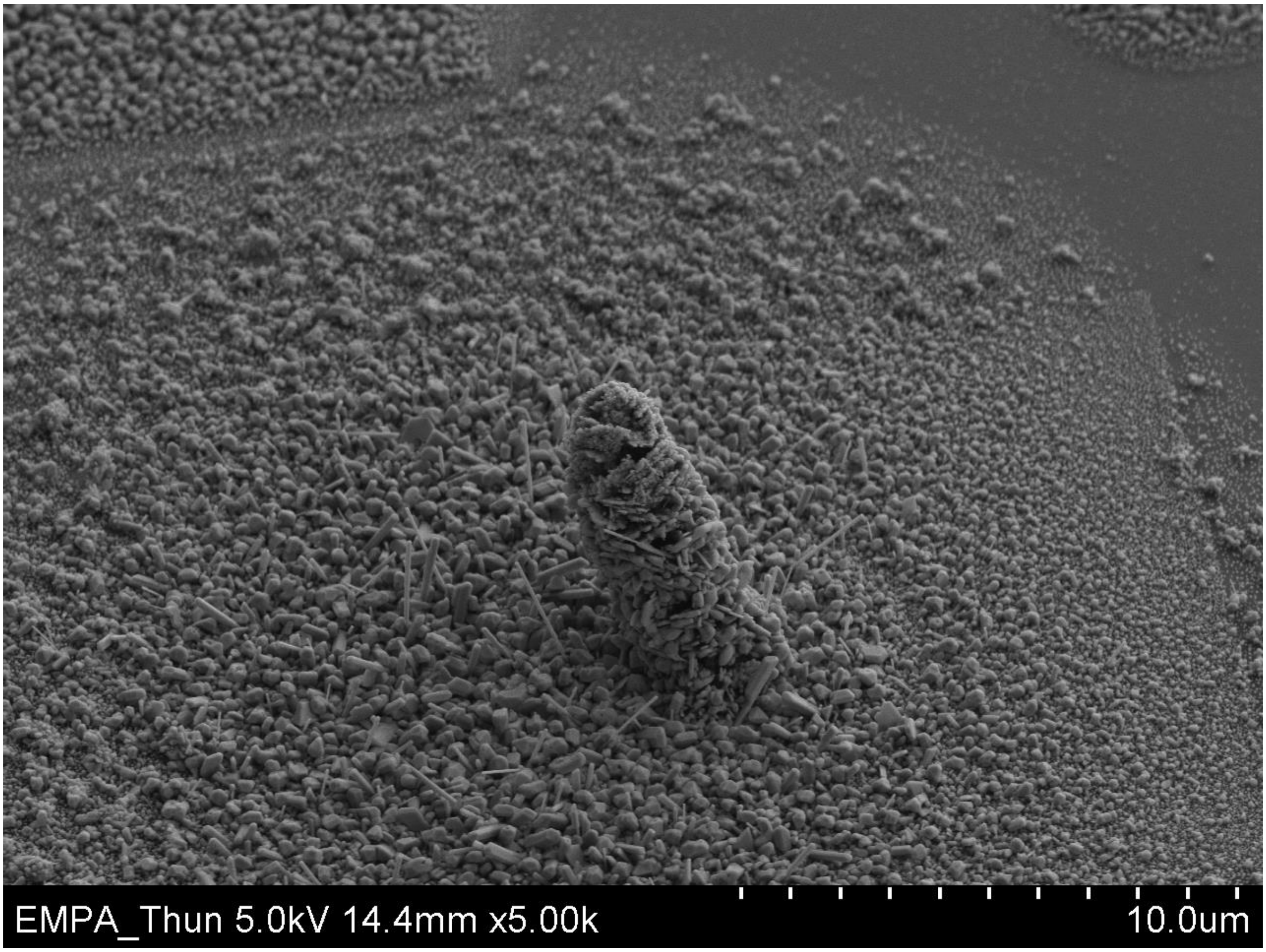

Figure S3.1. Pillar deposited using [$Ag_2(\mu\text{-}O_2CCF_3)_2$] as a precursor.

The deposit was made using 20 kV of acceleration voltage and I = 0.78 nA of beam current. Stage temperature was 186°C and the GIS temperature was 175°C. The deposit was made by constantly exposing single spot for 1 hour.

S4. Comparison between measured SAED patterns with Ag bulk standard

TEM measurements were performed as described in section 2.4 of the manuscript. The silver lattice parameters for all FEB deposits were similar. The average lattice parameter was equal to 3.949 ± 0.018Å and hence it was shorter than the value for bulk Ag 4.085 Å by about 3.3%. This is consistent with the literature trend shown for metallic silver nanoparticles [1].

S4.1. SAED pattern of [$Ag_2(\mu\text{-}O_2CCF_3)_2$]

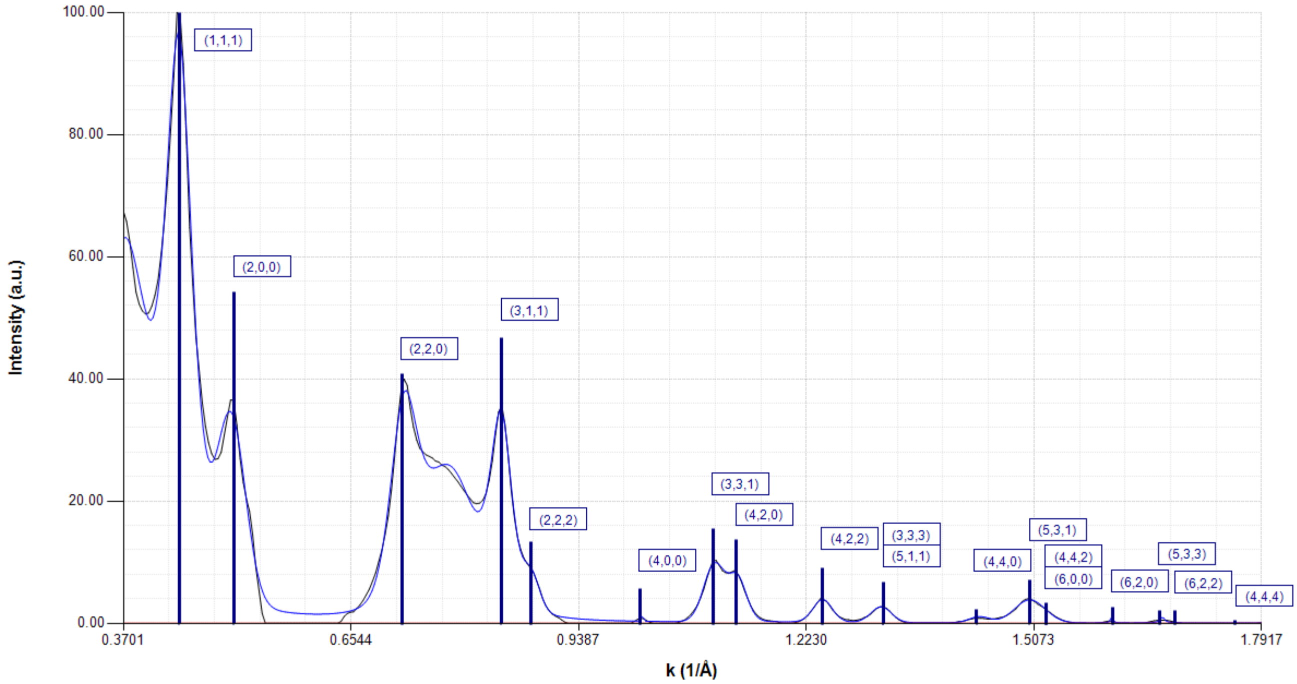

Figure S4.1. SAED pattern integrated around the diffraction ring, obtained from the FEB induced deposit with [$Ag_2(\mu\text{-}O_2CCF_3)_2$]. Comparison to pattern of pure Ag with peaks labeled (dark blue color bars and line).

Lattice parameter: 3.940 ±0.003 Å. The extra peak between Ag(220) and Ag(311) is not matched.

S4.2. SAED pattern of [$Ag_2(\mu$-$O_2CC_2F_5)_2$]

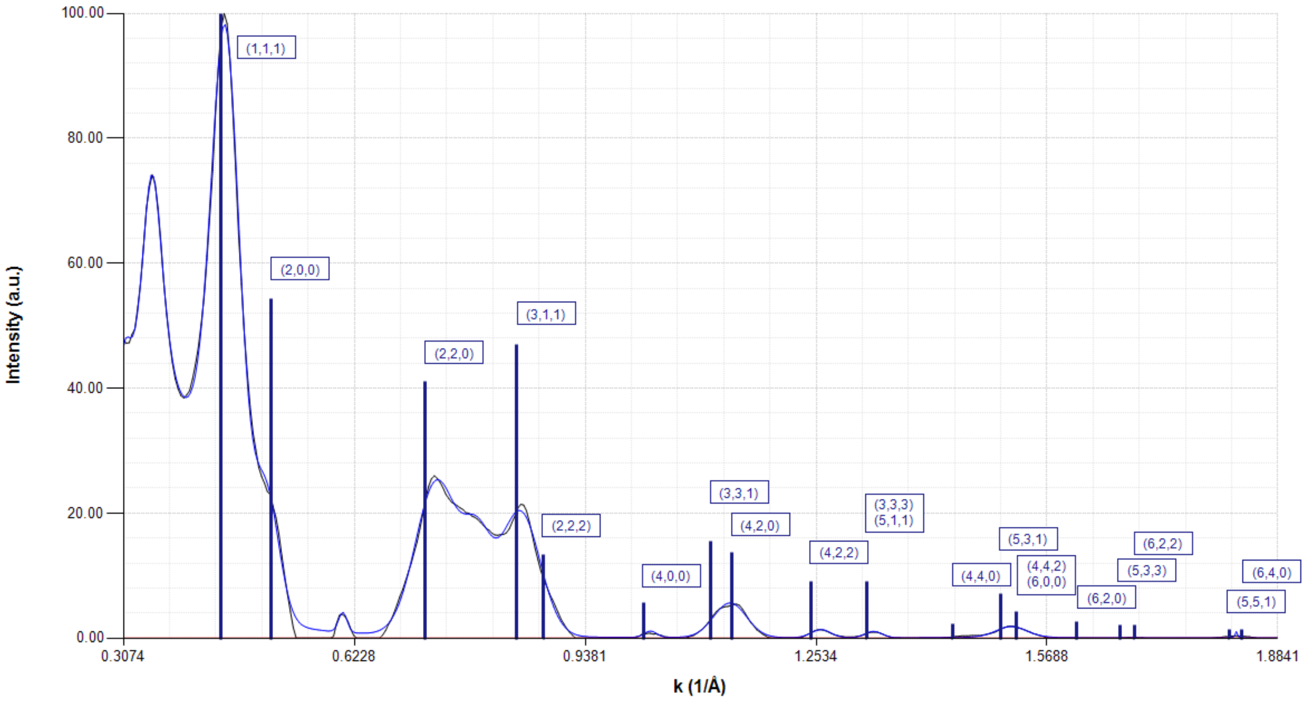

Figure S4.2. SAED pattern integrated around the diffraction ring, obtained from the FEB induced deposit with [$Ag_2(\mu$-$O_2CC_2F_5)_2$]. Comparison to pattern of pure Ag with peaks labeled (dark blue color bars and line).

Lattice parameter: 3.935 ±0.003 Å. The extra peak between Ag(220) and Ag(311) is not matched.

S4.3. SAED pattern of [$Ag_2(\mu\text{-}O_2CC_3F_7)_2$]

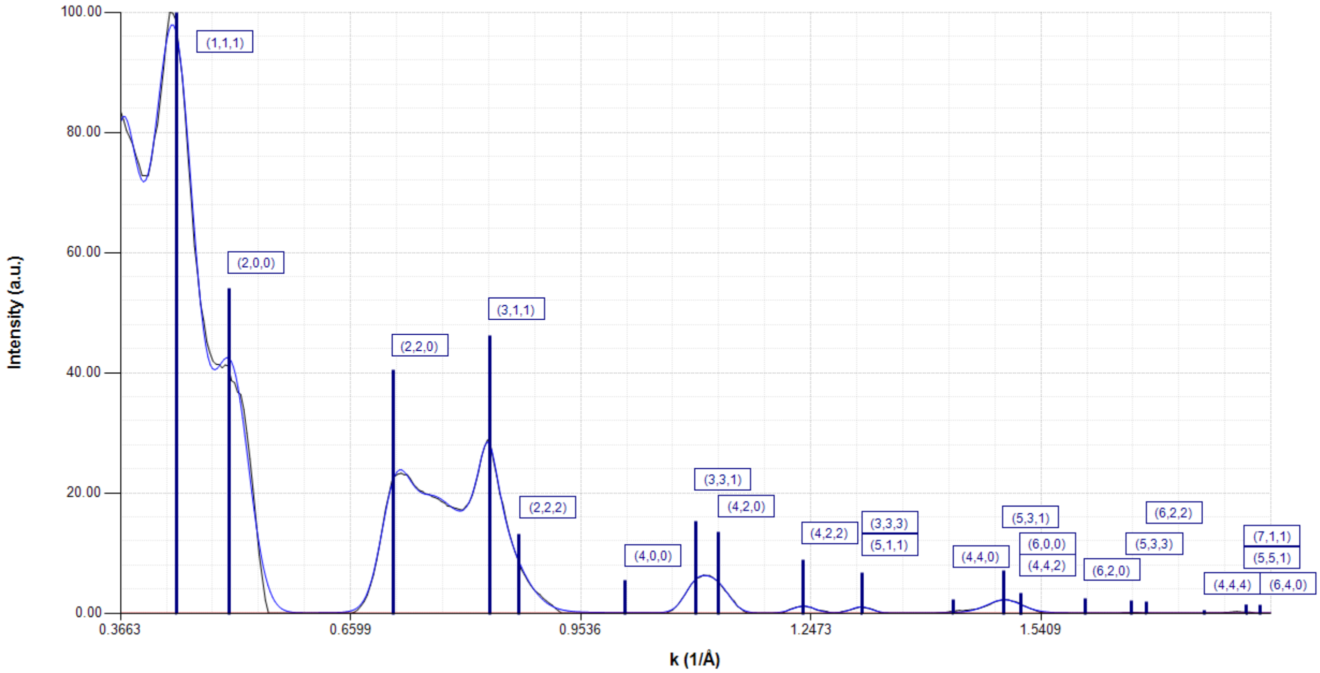

Figure S4.3. SAED pattern integrated around the diffraction ring, obtained from the FEB induced deposit with [$Ag_2(\mu\text{-}O_2CC_3F_7)_2$]. Comparison to pattern of pure Ag with peaks labeled (dark blue color bars and line).

Lattice parameter: 3.960 ±0.003 Å. The extra peak between Ag(220) and Ag(311) is not matched.

S4.4. SAED pattern of [Ag2(μ-$O_2C^tBu)_2$]

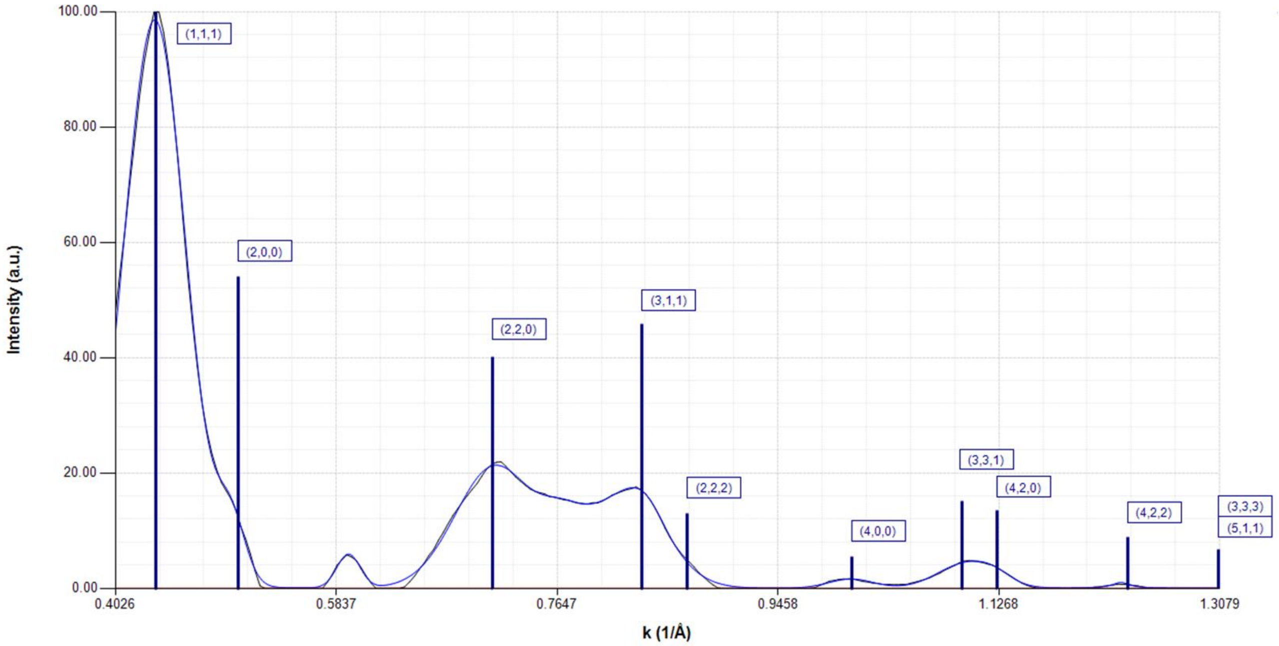

Figure S4.4. SAED pattern integrated around the diffraction ring, obtained from the FEB induced deposit with [$Ag_2(\mu\text{-}O_2C^tBu)_2$]. Comparison to pattern of pure Ag with peaks labeled (dark blue color bars and line).

Lattice parameter: 3.975 ±0.003 Å. The extra peak between Ag(220) and Ag(311) is not matched.

The distinctive extra peak between Ag(200) and Ag(220) is not from silver. It fits approximately to the (220) peak of cubic Ag_2O (4.718 Å). See figure S4.4.

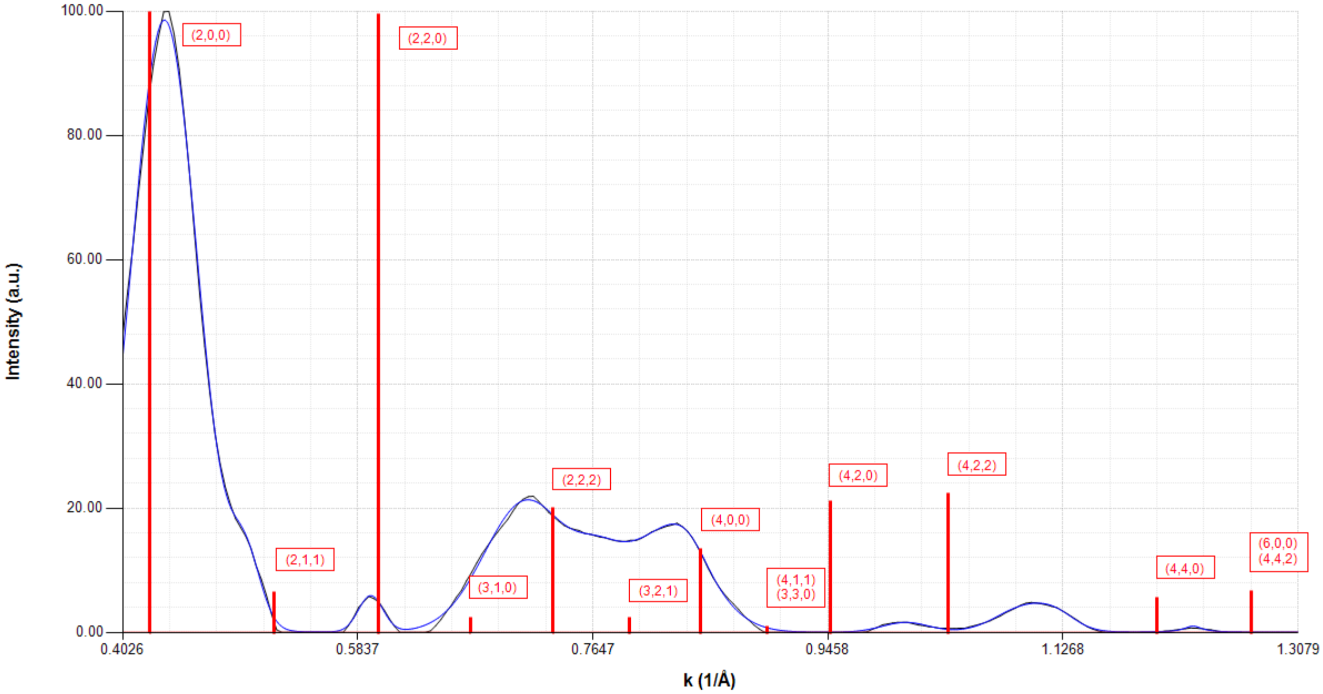

Figure S4.5. SAED pattern integrated around the diffraction ring, obtained from the FEB induced deposit with [$Ag_2(\mu\text{-}O_2C^tBu)_2$]. Comparison to pattern of Ag_2O with peaks labeled (red color bars and line).

S4.5. SAED pattern of [$Ag_2(\mu\text{-}O_2CC(Me)_2Et)_2$]

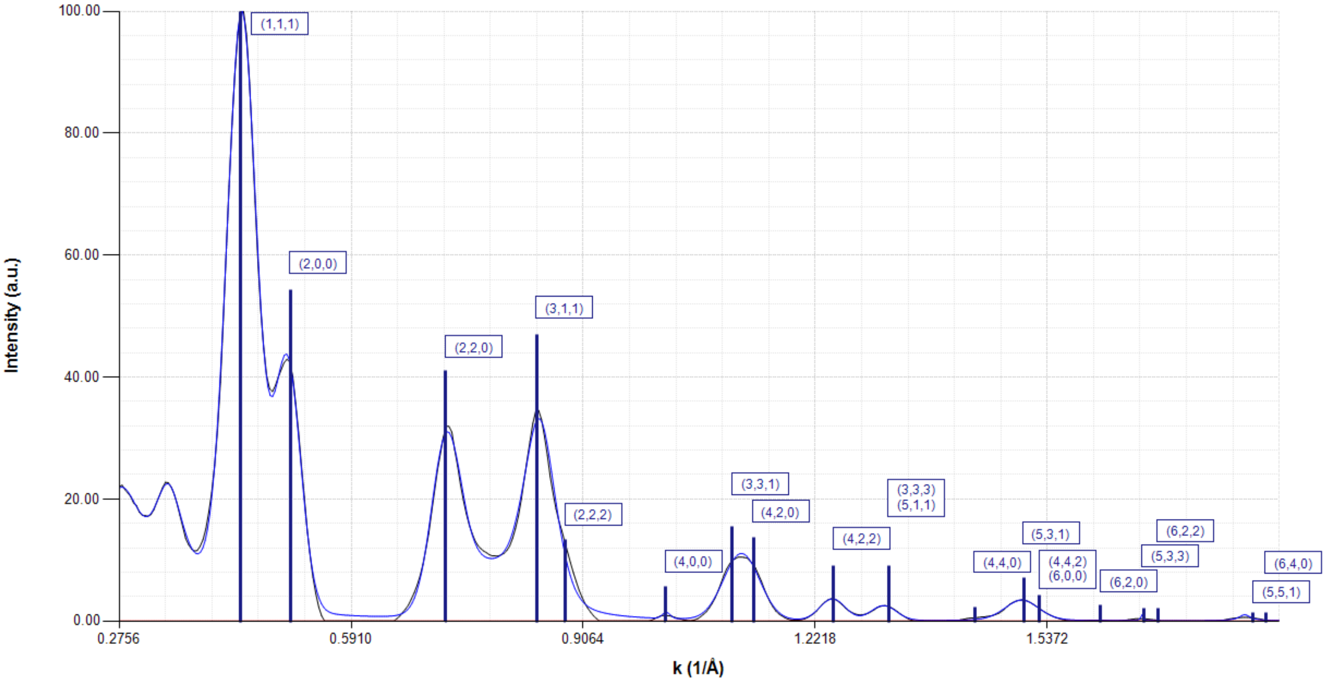

Figure S4.6 SAED pattern integrated around the diffraction ring, obtained from the FEB induced deposit with [$Ag_2(\mu\text{-}O_2CCMe_2Et)_2$]. Comparison to pattern of pure Ag with peaks labeled (dark blue color bars and line).

Lattice parameter: 3.935 ±0.003 Å.

References

1. Qi, W.H. and M.P. Wang, *Size and shape dependent lattice parameters of metallic nanoparticles.* Journal of Nanoparticle Research, 2005. **7**(1): p. 51-57.
